# Hypothyroidism and Diabetes-Related Dementia: Focused on Neuronal Dysfunction, Insulin Resistance, and Dyslipidemia

**DOI:** 10.3390/ijms23062982

**Published:** 2022-03-10

**Authors:** Hee Kyung Kim, Juhyun Song

**Affiliations:** 1 Division of Endocrinology and Metabolism, Department of Internal Medicine, Chonnam National University Medical School, 264 Seoyangro, Hwasun 58128, Korea; albeppy@chonnam.ac.kr; 2 Department of Anatomy, Chonnam National University Medical School, Hwasun 58128, Korea; 3 BioMedical Sciences Graduate Program (BMSGP), Chonnam National University, 264 Seoyangro, Hwasun 58128, Korea

**Keywords:** thyroid hormone, hypothyroidism, diabetes-related dementia, insulin resistance, dyslipidemia

## Abstract

The incidence of dementia is steadily increasing worldwide. The risk factors for dementia are diverse, and include genetic background, environmental factors, sex differences, and vascular abnormalities. Among the subtypes of dementia, diabetes-related dementia is emerging as a complex type of dementia related to metabolic imbalance, due to the increase in the number of patients with metabolic syndrome and dementia worldwide. Thyroid hormones are considered metabolic regulatory hormones and affect various diseases, such as liver failure, obesity, and dementia. Thyroid dysregulation affects various cellular mechanisms and is linked to multiple disease pathologies. In particular, hypothyroidism is considered a critical cause for various neurological problems—such as metabolic disease, depressive symptoms, and dementia—in the central nervous system. Recent studies have demonstrated the relationship between hypothyroidism and brain insulin resistance and dyslipidemia, leading to diabetes-related dementia. Therefore, we reviewed the relationship between hypothyroidism and diabetes-related dementia, with a focus on major features of diabetes-related dementia such as insulin resistance, neuronal dysfunction, and dyslipidemia.

## 1. Introduction

Thyroid hormones (THs) secreted from the thyroid gland are regulated by the thyrotropin-releasing hormone (TRH), which is released from the hypothalamus, and the thyroid-stimulating hormone (TSH), which is released from the pituitary gland [1]. Two forms of TH are commonly known as triiodothyronine (T3) and thyroxine (T4) [2]. T3 and T4 control many organs′ metabolic processes such as lipogenesis, liver function, glucose metabolism, and thermogenesis, through numerous cellular mechanisms and gene expression regulation; they do this by binding to TH receptors (TRs) [3,4,5]. In the central nervous system (CNS), THs have been known to promote neurogenesis, neuronal synaptic plasticity, synaptic transmission, modulation of neurotransmitters, and brain tissue repair systems [6,7].

TH dysregulation is directly associated with endocrine disorders and metabolic syndromes such as diabetes and obesity [8,9,10,11,12]. Hypothyroidism is referred to as decreased TH levels [13], characterized by impaired glucose metabolism such as reduction in glucose uptake and impaired energy metabolism, including decreased liver gluconeogenesis and reduced muscle gluconeogenesis and glycogenolysis [8]; therefore, it is associated with the development of diabetes [14,15]. In the CNS, TH dysfunction is strongly linked to the development of neurodegenerative diseases such as dementia [16,17]. On the other hand, some clinical studies mention that hypothyroidism is not directly related to dementia neuropathology, and that levels of TSH are not directly related to cognitive impairment [18,19]. Recent cohort studies have reported a negative correlation between TSH level and cognitive deficit caused by subcortical ischemic vascular dementia [20], as well as in the relationship between subclinical hypothyroidism and cognitive dysfunction in old people [21].

Subclinical and overt hypothyroidism contribute to mood dysregulation, anxiety, attention, psychomotor function, and cognitive impairment [22,23,24,25,26] and are highly linked to the development of neurological diseases such as Alzheimer’s disease and depression [27,28]. Patients with hypothyroidism showed hippocampal atrophy, leading to memory loss, cerebral blood flow impairment, and a reduction in working memory [29].

Clinical studies reported that subclinical hypothyroidism and overt hypothyroidism patients showed memory deficit due to hippocampus damage [23,30].

One functional MRI clinical study showed working memory loss in subclinical hypothyroidism patients, and subsequently, working memory loss was improved by thyroxine treatment [31]. Another clinical study suggested a reduction in verbal memory processing and hippocampal function in hypothyroidism patients [24,32].

Dementia is a global epidemic neurodegenerative disease. It affected approximately 47 million in 2015, and is increasing gradually worldwide [33]. In a recent global report, metabolic syndrome patients, such as those with type 2 diabetes, also suffer from dementia globally [34]. Diabetes patients showed neuropathological features such as cognitive impairment, amyloid beta accumulation, and reduced attention, which are involved in dementia pathologies [35]. Many researchers are investigating various risk factors and related mechanisms in diabetes-related dementia, because there are several common risk factors in type 2 diabetes (T2DM) and dementia [36,37,38].

Even though there are many risk factors related with the onset of type 2 diabetes and dementia, many researchers have suggested that diabetes patients are considerably involved in an increased risk for dementia [39,40]. Some epidemiological studies have mentioned that the risk of dementia and cognitive impairment is increased in patients with diabetes compared to subjects without [41,42]. The brain of a T2DM patient is found to exhibit brain atrophy, reduced brain volume, neuronal cell myelin loss and white matter vacuoles, leading to memory deficit [43,44,45].

Clinical studies suggest that patients with diabetes have more risk for dementia at RR 1.73 [46], for AD at RR 1.53 [41], and for vascular dementia at RR 2.27 [46] compared to patients without diabetes. Many researchers report that the relationship between diabetes and dementia are involved in vascular alteration [47,48] and impaired cerebral insulin signaling [49], leading to cognition [49,50].

A recent study mentioned the association between thyroid dysfunction and dementia and suggested related metabolic mechanisms [51]. Some studies report that the deficit of T3 aggravates mitochondrial dysfunction, poor glucose metabolism and impaired brain metabolic mechanisms, leading to cognitive impairment [52,53]. Other studies present evidence that the thyroid hormone modulates brain glucose metabolism, and subsequently contributes to memory formation in the brain hippocampus [54,55]. As such, thyroid dysfunction is strongly related to dementia and diabetic pathologies, such as insulin resistance and impaired glucose metabolism.

Therefore, TH is a major metabolic regulating hormone, and TH dysfunction leads to both systemic metabolic disorders, as well as neurological problems. However, its functions in diabetes-related dementia have not been fully understood until now. Thus, we review recent evidence on the relationship and related mechanisms between hypothyroidism and diabetes-related dementia.

## 2. TH Dysfunction in the CNS

THs are produced by the thyroid gland. They are secreted into the blood and contribute to the cellular mechanisms in many organs through blood circulation [56]. Thyroid dysfunctions are classified based on the serum levels of TH and TSH. Overt hypothyroidism manifests as decreased T3 and T4 levels and increased TSH; overt hyperthyroidism manifests as increased T3 and T4 levels and decreased TSH; subclinical hypothyroidism shows normal T3 and T4 levels with increased TSH and subclinical hyperthyroidism shows normal T3 and T4 levels and decreased TSH levels [57,58,59].

**Figure 1:**
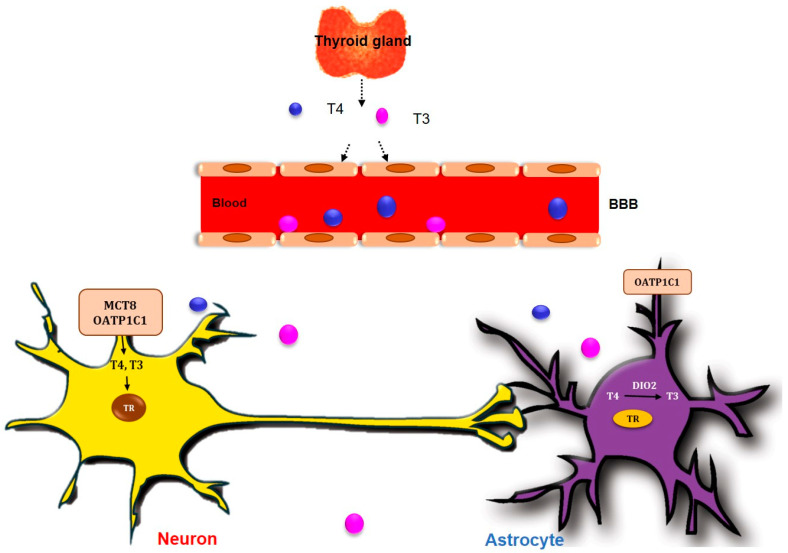
TH crosses blood brain barrier into the brain. T3 and T4, secreted from thyroid gland, cross the blood–brain barrier (BBB) into the brain through MCT8 and OATP1C1 TH transporters in neuron and OATP1C1 TH transporters in astrocytes. T4 is converted to T3 by type II 5′-deiodinase enzyme (*DIO2*) in astrocyte.

The concentration of T4 and T3 in the CNS is known to be approximately 20% that of blood serum [60]. TH is important for nervous system development and the maintenance of brain function, including the cognitive function process and affective mood process [57,61]. For the TH to enter into the brain, T4 and T3 cross over the blood brain barrier (BBB) of the choroid plexus through an MCT8 TH transporter or OATP1C1 TH transporter [62] (Figure 1). T4, as a prohormone, is taken up into astrocytes through OATP1C1, and it should be converted into T3 through an enzymatic reaction of deiodinase enzymes to work in the cells [63]. Deiodinase 2 (*DIO2*) is known to be the main enzyme responsible for the conversion of T4 into functional T3 (fT3) in the brain [64] (Figure 1). Subsequently, T3 generated from astrocytes enters the neuronal cells through an MCT8 transporter [65] (Figure 1). T3 binds to the nuclear receptor TRs and leads to a nucleus transcriptional change in CNS cells [62,66] (Figure 1). There are three TR isoforms: alpha and two forms of beta [67,68]. TRα1 is commonly expressed in the brain and skeletal muscle, and is existed over 80% of all TR expression in the brain [69,70]. T3 contributed to the progression of neural stem cells for memory formation, neuronal plasticity, and brain development in the subventricular zone and hippocampus dentate gyrus by binding TRα in a hypothyroid animal model [71,72]. TRβ1 is more prevalent and is expressed in the brain, heart, liver, and kidney, and TRβ2 is concentrated in pituitary and hypothalamic tissues [73].

The imbalanced secretion of neurotransmitters such as acetylcholine, and noradrenaline in the hippocampus and prefrontal cortex, is routinely observed in hypothyroid and hyperthyroid animal models [74,75]. Furthermore, thyroid dysfunction leads to the imbalanced secretion of neurotransmitters, such as γ-aminobutyric acid (GABA), serotonin, catecholamine, and neurite growth factors in the brain, ultimately leading to cognitive impairment and depression [57,74,76].

Several clinical studies have shown that there is a strong, positive correlation between hypothyroidism and memory loss, attention error, poor verbal ability, abnormal motor function, deafness, and spasticity [23,24,77,78]. Patients with hypothyroidism showed typical behavioral symptoms similar to those of depressive and anxiety disorders [79,80,81,82]. The treatment of levothyroxine (L-T4) in patients with hypothyroidism improves cognition, mood disorders, and behavioral problems [83,84,85]. Subclinical and overt hyperthyroidism leads to apathy, lethargy, and depressive mood disorders [86,87].

One clinical study revealed that higher levels of T3 are found in the brain hippocampus and cerebrospinal fluid of Alzheimer’s disease patients [88]. Another study showed the negative correlation between T3 level and the development of Alzheimer’s disease [89]. One study reported that the administration of T3 offers protection to the prefrontal cortex, hippocampus, and amygdala—which are related to cognition and emotion—against amyloid beta toxicity in Alzheimer’s disease [90].

In this review, we summarized the common mechanisms and specific noteworthy risk factors between hypothyroidism and diabetes-related dementia pathologies.

## 3. Diabetes-Related Dementia and THs

Dementia, as a progressive metabolic disease, impairs memory function gradually [91]. Several studies have reported that type 2 diabetes and hyperlipidemia showed an increased risk of dementia such as vascular dementia, Alzheimer’s disease, and cognitive dysfunction [42,92,93,94,95,96]. In patients with Alzheimer’s disease, metabolic imbalance, such as central and peripheral insulin resistance and impaired insulin signaling, were observed [39,97,98].

Poor insulin signaling, insulin resistance, and oxidative stress condition caused by diabetes contribute to neuronal degeneration, leading to memory loss [99]. Previous studies have shown that insulin resistance aggravates tau hyperphosphorylation and the formation of neurofibrillary tangles, involving memory loss [100,101]. Neuroinflammation and impaired cerebral vasculature, leading to cognitive decline, are a common feature in dementia and the diabetic brain [102,103]. Furthermore, dyslipidemia related to apolipoprotein E (*APOE*) e4 alleles, caused by diabetes, leads to lower cognitive performance, lower attention and lower motor function [104]. Some studies have reported that diabetes animal models showed reduced neurogenesis [105], mitochondrial dysfunction in neuronal cells [106], and synaptic failure in the hippocampus [107], which is involved in learning and memory [107]. Furthermore, insulin resistance in metabolic syndromes leads to an imbalanced secretion of neurotransmitters such as acetylcholine [101,108], dopamine [109,110], glutamate [111], and 5-HT serotonin [112], leading to cognitive dysfunction [113].

Several studies have reported that elevated TSH level are strongly associated with insulin resistance [114] and hyperlipidemia [115]. Subclinical hypothyroidism is easily observed in patients with type 2 diabetes compared with normal subjects [13,116,117].

TSH level is related with diabetic peripheral neuropathy [118], and one study has shown that TSH and TSH receptor administration aggravates diabetic peripheral neuropathy by inducing apoptotic responses in Schwann cells [119].

TH controls the function and cellular homeostasis of many organs, such as the liver, heart, adipose tissue, liver, and brain, and modulates energy metabolism, free fatty acid oxidation, lipid metabolism, and the thermogenesis process [67,120,121]. T3 promotes metabolic enzymes such as acetyl-CoA carboxylase, and influence fatty acid synthesis and fatty acid oxidation [68,122]. Previous studies have shown that T3 decreases the circulation of free fatty acids and triglycerides in metabolic imbalance animal models [123,124]. T3 promotes several transcription factors related with lipogenesis, such as carbohydrate-responsive element-binding protein [125], and TSH promotes lipogenesis-related transcription factors such as the peroxisome proliferator-activated receptor-gamma (PPAR-γ) and the sterol regulatory element-binding transcription factor 1 [126]. Moreover, TSH decreases phosphorylation of 3-hydroxy-3-methylglutaryl coenzyme A (HMG-CoA) reductase [127].

Considering the associated previous consequences of diabetes-related dementia pathologies and thyroid dysfunction, we need to conduct further studies to understand the mechanism of TH in the CNS. Collectively, we summarize each point regarding the relationship between hypothyroidism and diabetes-related dementia.

## 4. Neuronal Cell Damage and Imbalanced Neurotransmitters in Hypothyroidism

**Figure 2:**
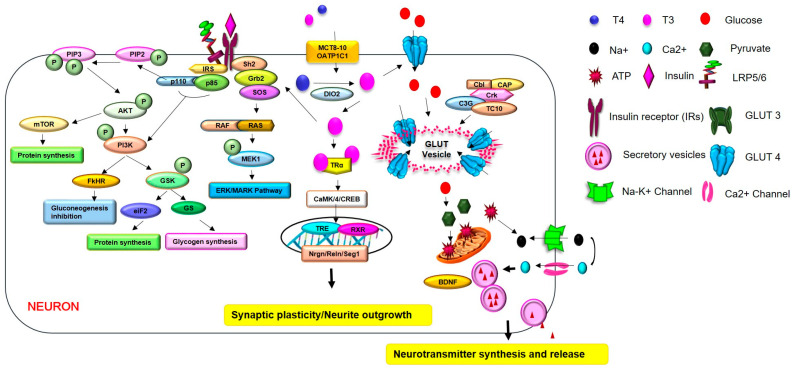
TH regulates glucose metabolism and the secretion of neurotransmitters in hypothyroidism. TH controls glucose uptake and insulin sensitivity by maintaining the expression of glucose transporters such as glucose transporter (GLUT) in CNS cells. TH prevents the cell death process and promotes neurite outgrowth and synaptic plasticity through several genes, such as the *Nrgn*, *Reln* and *Srg1* genes, and through the CaMK/4/CREB signaling in neuron. TH modulates the expression of neurotransmitters, including dopamine, glutamate, GABA, and BDNF, through several kinds of signaling, and can ultimately control brain functions. Additionally, TH increases the activation of PI3K/Akt signaling, and GSK3β signaling is related to the enhancement of insulin sensitivity. TH boosts the expression of *BDNF*, leading to the activation of PI3K/Akt signaling, which is involved in cognitive function. Finally, the improvement of insulin sensitivity leads to the enhancement of cognitive function. In hypothyroidism, reduced levels of TH lead to impaired synaptic plasticity, cognitive deficit, abnormal neurotransmitter release, impaired neurite outgrowth, and insulin resistance.

T3 prevents the cell death process of primary cortical neurons in hypoxia apoptosis conditions by increasing anti-apoptotic gene expression and phosphorylation of CaMK/4/CREB signaling [128,129,130,131,132,133]. Additionally, T3 could control neuronal outgrowth and synaptic plasticity through the regulation of the neuronal-specific gene *Nrgn* [134,135]. The neuronal-specific gene *Nrgn* is markedly expressed in hippocampal neuronal dendritic spines, and regulates spatial memory formation and anxiety-like behaviors [136]. Other studies have shown that T3 administration contributes to brain development by increasing *Reln* gene expression [137,138]. The *Reln* gene induces neuronal migration in the neocortex and promotes neurogenesis and synapse formation by binding with apolipoprotein E receptors [139,140] (Figure 2).

During brain development, TH induces synaptic plasticity by increasing spine cytoskeleton formation and microtubule reorganization through synaptotagmin-related gene 1 (*Srg1*) gene expression [141,142]. TH controls microtubule-related proteins and Tau proteins in neurons during brain development [143,144]. Additionally, TH controls neuronal filaments in neurons and glial fibrillary acidic protein filament in astrocytes [130]. TH promotes neuronal migration, neuronal differentiation, and glia maturations [145,146,147]. Based on this evidence, hypothyroidism leads to neuronal cell damage and reduced neurogenesis and glia activation.

Hypothyroidism leads to dopaminergic neuronal loss [148], because T3 and T4 could maintain dopaminergic neurons by increasing *Nurr1* expression, and protect neuronal damage by regulating dopamine levels in the CNS [149] (Figure 2).

T3 could regulate neurotransmitter glutamate uptake in astrocytes [150], reduces N-methyl-d-aspartate-evoked currents in the hippocampal neuron, and inhibits glutamate-induced neuronal cell damage in hippocampal neurons [151] (Figure 2).

TH controls the maturation of GABAergic neurons [152]. It could regulate the release and uptake of GABA in neuronal synaptosomes from the cerebral cortex [153] and affects GABA A receptors in the cerebral cortex [154]. Therefore, if hypothyroidism occurs during brain development, neuronal cell differentiation and neuronal cell proliferation in the cortex could be impaired [155,156] (Figure 2). Other studies have found that hypothyroidism leads to the decreased expression of brain-derived neurotrophic factor (*BDNF*) as a neurotrophic factor in the hippocampal brain regions [138,157] (Figure 2).

Hypothyroidism could damage neuronal cell death and aggravate synaptic plasticity, as well as promote the abnormal release and uptake of neurotransmitters such as dopamine and GABA in the brain, related to neuropathologies.

## 5. Brain Insulin Resistance in Hypothyroidism

Insulin, a peptide hormone produced by the pancreas, regulates glucose levels and affects various cellular mechanisms [158]. In the CNS, insulin increases brain energy metabolism and induces hippocampal neurons for memory formation [159,160]. Brain insulin resistance is considered the impairment of insulin activity and aggravates neuronal cell death, leading to memory loss and cognitive impairment [159,161,162,163].

Insulin regulates amyloid beta accumulation and tau hyperphosphorylation and glycogen synthase kinase 3 (GSK3) activity, leading to neuronal cell damage and memory dysfunction [164]. GSK3β signaling, the downstream pathway of insulin signaling, contributes to tau hyperphosphorylation. It is related with microtubule destabilization of neurons, and leads to cognitive decline [165,166]. The activity of GSK3β signaling is regulated by TH activity [167] (Figure 2). Further, BDNF as a neurotrophic factor boosts the activation of PI3K/Akt signaling and insulin signaling in CNS cells [168]. Insulin promotes the secretion of BDNF, which promotes neurite length, synaptic formation, and neuronal cell survival, and ultimately improves depressive mood disorder and Alzheimer’s disease pathologies [169,170]. Several studies have reported that T3 was shown to increase the expression of BDNF in the hippocampus region [138,157] (Figure 2). The reduction in BDNF expression leads to the decreased expression of synaptic plasticity, related genes such as calcineurin and cAMP response element-binding protein (*CREB*), after thyroid gland removal surgery [171].

One study mentioned that the T3/reverse T3 ration is increased in insulin resistance status, and thyroid hormone function affects insulin resistance condition [172,173]. Insulin upregulates *DIO1* and *DIO2* activity and contributes to the conversion of T4 into functional T3 [174,175].

Another study indicated that high levels of TSH are associated with more obese status and increased metabolic profiles such as that of insulin resistance [176].

A recent meta-analysis reported that abnormal levels of functional T3 and T4 are related to the onset and development of type 2 diabetes (T2DM) [177].

Patients with diabetes showed a higher risk of memory loss, neuropathological problems, and prevalence of dementia [178,179]. Moreover, Alzheimer’s disease dementia patients showed impaired insulin signaling in the brain, which is considered an early stage of cognitive deficit [180].

TH strongly contributes to glucose metabolism and insulin resistance [114,168,181,182,183,184,185]. A recent study showed that a diabetes animal model exhibits hypothyroidism, increased inflammatory responses, and reduced insulin sensitivity [186]. Additionally, in clinical studies, patients with hypothyroidism displayed increased metabolic syndrome, and poor glycemic control and insulin resistance [14,187,188].

Considering a previous study, T3 and T4 exhibit genomic effects by regulating Ca++ entry into cells, as well as the activation of some kinases [189]. Phosphoinositide 3-kinase (PI3K) is the one of TH-activated kinases, and activated PI3K signaling contributes to insulin signaling [190]. Some studies have demonstrated that TH increases Akt phosphorylation related to cognitive function in hippocampal neurons, and subsequently enhances insulin sensitivity [191,192,193] (Figure 2). TH activity is associated with glucose metabolism and insulin signaling through the regulation of glucose transporter expression [168,185,194]. One in vitro study mentioned that TH controls glucose uptake into cells through Glucose transporter 1 (GluT1), and modulates insulin secretion [195] (Figure 2).

TH could control cognitive function by modulating insulin activity and type 3 deiodinase activity [196]. Additionally, a recent study reported that polymorphism of TH-activation-related type 2 deiodinase enzyme is related with insulin resistance [197]. Furthermore, some studies have mentioned that TRs and the type II 5′-deiodinase enzyme (*DIO2*) were high in several brain regions such as the hippocampus and cortex, which are related to memory function [64], and decreased TRs were observed in the brains of Alzheimer’s disease patients [198].

Considering that brain insulin resistance is a critical factor in diabetes-related dementia, the relationship between hypothyroidism and diabetes-related dementia is an important issue.

## 6. Dyslipidemia in Hypothyroidism

A previous study shows that dyslipidemia is linked to both type 2 diabetes and Alzheimer’s disease [199]. Blood–brain barrier (BBB) disruption is easily observed in dementia, such as in Alzheimer’s disease [200] and diabetes [201]. The increased release of cholesterol and the impaired cholesterol transport in neurons were observed in dementia brains [202,203].

Lipid contributes to BBB function and BBB integrity, and has a role in the development of Alzheimer’s disease pathology [204]. Some studies have reported that elevated triglycerides are strongly related to dementia development [205,206].

In the brain, high cholesterol leads to increased amyloid beta production from the amyloid beta protein precursor [207], and reduced soluble amyloid precursor protein production [208]. Some studies have demonstrated that cholesterol intake accelerates amyloid beta deposition in the brain [204,209]. Other studies have reported that amyloid beta 42 formation and tau hyperphosphorylation were modulated by cholesterol [210].

ApoE, as a major constituent of chylomicrons in blood circulation, is the most abundant apolipoprotein in the brain, and is associated with cholesterol transfer, amyloid beta accumulation, higher plasma concentrations of low-density lipoprotein (LDL) cholesterol, and higher risk of Alzheimer’s disease dementia [211]. One study reported that many patients with dementia have at least one *ApoE* ε4 allele, compared to healthy subjects [212].

Several studies have identified that patients with diabetes showed severe hyperlipidemia [63] and dementia neuropathology [213,214]. One cohort study reported that diabetes leads to hyperlipidemia and high risk of dementia [215].

Dyslipidemia (such as hypercholesterolemia), low high-density-lipoprotein (HDL) cholesterol, high prevalence of small low-density-lipoprotein (LDL) cholesterol particles, high LDL cholesterol, and hypertriglyceridemia contribute to cognitive impairment [216,217], and lead to an increased risk for Alzheimer’s disease dementia [218,219,220,221,222,223].

**Figure 3:**
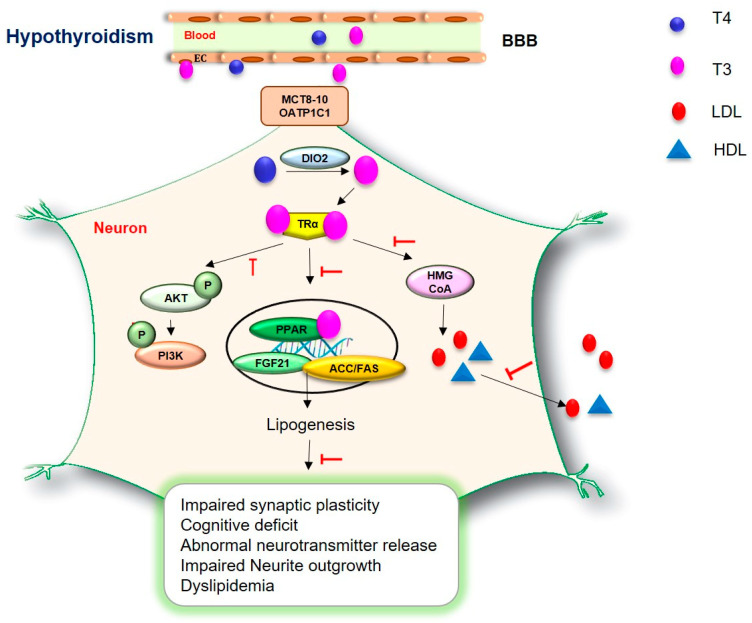
TH regulates dyslipidemia in hypothyroidism. TH modulates the secretion of cholesterol, the expression of HMG-CoA reductase, and the synthesis of LCL-cholesterol and HDL-cholesterol. Additionally, TH controls the expression of the acetyl-CoA carboxylase and fatty acid synthase (ACC/FAS) ACC/FAS gene involved in lipid metabolism. TH promotes the expression of the *FGF21* gene and activates proliferator-activated receptor α (PPARα) signaling, which is related to triglycerides. In hypothyroidism, reduced levels of TH lead to impaired synaptic plasticity, cognitive deficit, abnormal neurotransmitter release, impaired neurite outgrowth, and dyslipidemia.

TSH could modulate the secretion of serum cholesterol, the expression of HMG-CoA reductase, and the synthesis of LCL-cholesterol [224] and HDL-cholesterol [225] (Figure 3). T3 regulates the expression of the acetyl-CoA carboxylase and fatty acid synthase (ACC/FAS) ACC/FAS gene involved in lipid metabolism [226] (Figure 3). TSH regulates HMG-CoA by stimulating the expression of the *SREBP-2* gene [227].

Hypothyroidism contributes to various functions in the regulation of serum lipid profiles [228]. A previous study mentioned that hypothyroidism could decrease liver uptake of free fatty acid, reduce cholesterol secretion, and decrease plasma triglyceride clearance, accompanied with reduced lipoprotein levels [229,230,231]. Hypothyroidism leads to lower energy expenditure and high lipid storage [232,233]. Moreover, hypothyroidism has revealed lower plasma cholesteryl ester transfer proteins and decreased very-low-density lipoproteins [234].

Hypothyroidism has shown severe hyperlipidemia, such as the reduction in cholesterol synthesis, by reducing the expression of β-hydroxy β-methylglutaryl-*CoA* (HMG-CoA) reductase in the liver [63] (Figure 3).

A current study suggested that patients with subclinical hypothyroidism showed high levels of total cholesterol and abnormal lipid profiles [235]. One clinical study demonstrated that more than 30% and more than 90% of patients with hypothyroidism showed increased total cholesterol and increased LDL cholesterol levels and dyslipidemia, respectively [63,236,237]. Other recent studies have shown that patients with hypothyroidism manifested with hypertriglyceridemia and exhibited elevated triglycerides and triglyceride-rich lipoproteins in the serum [238,239]. A previous study showed that patients with hypothyroidism exhibited higher levels of LDL cholesterol and homocysteine [240].

A recent study reported that fibroblast growth factor 21 (FGF21), which is a cytokine increased in dyslipidemia [241], was reduced in hypothyroidism patients compared to normal subjects [242]. T3 induces the expression of the FGF21 gene and activates proliferator-activated receptor α (PPARα) signaling, which is related to triglycerides [243] (Figure 3). The present study reported that patients with hypothyroidism exhibit low HDL-cholesterol, elevated homocysteine, and increased apolipoprotein ApoA 1 concentration [244]. Given that dyslipidemia is a crucial issue in diabetes-related dementia, the association between dyslipidemia and hypothyroidism warrants further investigation for finding specific treatment for diabetes-related dementia.

## 7. Neurological Problems in Hypothyroidism

TH dysfunction causes numerous neurological problems, including anxiety, depression, and cognitive impairment [55]. In dementia patients, the positive relationship with TH dysfunction has been observed in some studies [245,246]. Several studies have identified that hypothyroidism changes neuronal function and is involved in cognition processes [247,248]. The study suggested that the hippocampus, as the central brain region of cognitive performance [249], was damaged, and apoptotic processes were found in the brains of patients with hypothyroidism [250]. Other recent studies reported that hypothyroidism exhibits an increased inflammatory response in hippocampal brain regions and induces spatial memory loss [251,252]. The subclinical hypothyroidism animal model exhibits spatial memory loss through BDNF TrkA/p75NTR signaling pathway [253]. One animal study reported that hypothyroidism damages learning and memory function, aggravates long- and short- term memory, and impairs synaptic plasticity [254]. Another study showed that hypothyroidism was noted in elderly people with mild cognitive impairment [255]. Previous studies reported that hypothyroidism brains showed disruption of synaptic plasticity, which impaired long-term potentiation in the hippocampal CA1 region [256,257,258,259]. Based on previous studies regarding the effect of THs on cognitive impairment and depressive disorders, the neurological features of both hypothyroidism and diabetes-related dementia warrant further study to understand the cognitive decline in both hypothyroidism and diabetes-related dementia.

## 8. Conclusions

Thyroid dysregulation is an important issue both in metabolic syndromes and neurological disorders, because TH is involved in the regulation of cell death response, the modulation of neuronal function, the production of neurotransmitters, the regulation of glucose metabolism, and the regulation of lipid metabolism. Here, we summarized the mechanisms of neuronal dysfunction, insulin resistance, and dyslipidemia in both hypothyroidism and diabetes-related dementia. We suggest that further studies should be conducted on the correlation and mechanism between hypothyroidism and diabetes-related dementia, due to the association with the multiple metabolic processes of THs.

Thus, we assume that the modulation of insulin action and triglyceride levels will be beneficial for the treatment of both hypothyroidism and diabetes-related dementia.

Given the existing clinical approach to thyroid medication use in Alzheimer’s disease patients [260], appropriate monitoring of TH levels would be a good approach to predicting neuropathological problems caused by diabetes-related dementia in advance; modulation of TH levels would be another approach to improve neuropathogenesis in diabetes-related dementia.

## Abbreviations

Thyroid hormone, TH; thyroid stimulating hormone, TSH; triiodothyronine, T3; tetraiodothyronine, T4; thyroid hormone receptor, TR; central nervous system, CNS; carbohydrate-responsive element-binding protein, ChREBP; peroxisome proliferator-activated receptor-gamma, PPAR-γ; sterol regulatory element-binding transcription factor 1, SREBP-1c; N-methyl-d-aspartate, NMDA; apolipoprotein E, APOE; free thyroxine, FT4; free triiodothyronine, FT3; glial fibrillary acidic protein, GFAP; synaptotagmin-related gene 1, Srg1; glycogen synthase kinase 3, GSK3; brain-derived neurotrophic factor, BDNF; blood–brain barrier, BBB; cholesteryl ester transfer proteins, CETPs; very-low-density lipoproteins, VLDL; low-density lipoprotein, LDL; high-density lipoprotein, HDL; β-hydroxy β-methylglutaryl-*CoA,* HMG-CoA; fibroblast growth factor 21, FGF21; triglyceride, TG.
